# Continuous bubble-free laser printing of plasmonic nanostructures enabling annealing-free ohmic conduction and multifunctional trapping/spectroscopy studies

**DOI:** 10.1039/d5na00742a

**Published:** 2025-10-29

**Authors:** K. Monisha, Bharath Bannur, Shreyas M. S., Sajan D. George

**Affiliations:** a Department of Physics, Government First Grade College Sullia Karnataka 574239 India; b Manipal Institute of Applied Physics, Manipal Academy of Higher Education Manipal 576104 India; c Centre for Applied Nanosciences (CAN), Manipal Academy of Higher Education Manipal 576104 India sajan.george@manipal.edu

## Abstract

Direct optical printing of nanoparticles shows tremendous potential as it enables the fabrication of arbitrary plasmonic patterns and affordable designs for diverse applications. Although significant attention has been paid to the bubble-assisted printing of nanoparticles, the requirement for complex arrangement, tedious optimization processes, high laser power, and double-hump printing profile make it a less favorable methodology as compared to the bubble-free printing of particles from colloids. Thus, a bubble-free approach to the optical patterning of plasmonic nanoparticles is highly desirable. Here, we demonstrate a laser-assisted, bubble-free optical patterning method for plasmonic nanoparticles directly from a colloidal solution. Furthermore, we investigated the role of laser writing speed and power in the printed plasmonic pattern. Interestingly, the *I*–*V* characteristics of the printed patterns exhibit ohmic conduction without the need for post-annealing, highlighting the potential of printed metallic nanoparticles for microelectronic applications. As a proof of concept, the fabricated conducting circuit *via* printing is used for the electrothermal trapping of biological cells. In addition, the spectroscopic applications of the printed plasmonic patterns are explored *via* surface-enhanced Raman spectroscopy, with crystal violet as a probe molecule. Through the optimized direct optical printing, we demonstrate the optical printing of metallic nanoparticles that exhibit excellent electrical conductivity and Raman scattering signal enhancement, thus providing a versatile platform for multi-analytical approaches.

## Abstract 2

A laser-assisted, bubble-free optical printing of plasmonic nanoparticles from the colloid is demonstrated, and the printed conducting patterns are used for electrothermal trapping of objects and surface-enhanced Raman spectroscopy applications.

## Graphical abstract

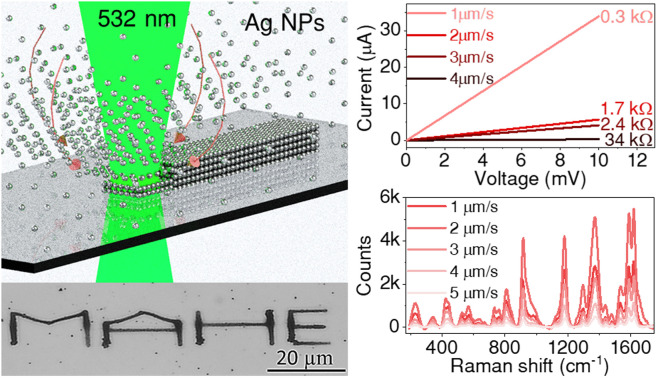

## 1. Introduction

The commonly adopted nanofabrication strategies rely on sophisticated lithographic approaches or chemical synthesis.^1,2^ However, the popular photolithographic technique suffers from intricacies of a multi-step process whereas e-beam and ion-beam lithography are not economical for scaling up applications.^3,4^ On the other hand, the chemical route that can allow the printing or patterning of nanoparticles, including plasmonic nanoparticles *via* various techniques such as self-assembly,^5^ the Langmuir–Blodgett method,^6^ polymer pen lithography,^7^ contact printing,^8^*etc.* is now increasingly used for nanodevice fabrication. Fabricating superstructures from plasmonic nanoparticles from colloidal solution *via* directed assembly is a subject of immense research as it can create functional materials and devices.^9^ Along these lines, directed assembly of colloidal materials has been attempted *via* external stimuli such as light, and electric, acoustic, and magnetic fields.^10^ Amongst these, optically directed light assembly or printing of plasmonic nanoparticles is of high importance as it can find applications in metamaterials, photonics, spectroscopy, and nanodevices.^11,12^ Early studies have shown that the optical tweezer technique, wherein the gradient force generated through a highly focusing optical element balances the optical scattering force along the propagation direction can trap and assemble the particles from the colloidal solution.^13–16^ However, the complexity and necessity of superior laser beam quality and high numerical aperture objectives to manoeuvre the ultra-small particles, including plasmonic nanoparticles, demanded exploring an alternate light-assisted approach to trap and print the particles.^17,18^ In addition, high intensity due to the focusing of light through the high NA objective can damage the patterning particle.^19^ Recently, optoelectronic tweezers where optically patterned electrodes on a photosensitive substrate are used to grab and immobilize the particles on the surface of hydrogenated amorphous silicon are also explored.^20^

Lately, light-induced thermal or electrical field gradients have been exploited to assemble colloidal particles precisely.^11,20–25^ In particular, focusing higher optical radiation from a laser source onto a light-absorbing metallic substrate can locally increase the temperature leading to solvent evaporation and formation of microbubbles with a strong thermal gradient.^26^ The thermal gradient can bring the particles towards the bubble and the Marangoni convection that originates due to the stress tension gradient at the bubble interface allows accumulation of particles at the bubble interface.^27–30^ Through the hydrophobic interactions of the bubble at the triple-contact line with the interface, the particles are immobilized at the interface.^29^ This technique has been effectively employed to print plasmonic nanoparticles and 2D materials like MXenes.^30–32^ However, these methods require a pulsing technique to make the pattern continuous and post-processing such as a laser, chemical, or oven sintering process to make it conduct.^26,33^ In addition, the bubble-assisted printing technique still suffers from the intricacy and dependence of surface chemistry.^26^ Importantly, the double-hump patterning resulting from radial symmetry presents a challenge to uniformity.^34^ Recently, we illustrated the direct printing of plasmonic nanoparticles from the colloidal solution without the usage of a light-absorbing substrate and microbubble formation.^22,23^ In this approach, optical radiation along the propagation direction is absorbed by the plasmonic nanoparticles that lead to a temperature gradient along the propagation direction and consequent Marangoni flow. The Marangoni flow brings the particles towards the irradiation interface wherein the particles are immobilized due to van der Waals interaction. The potential of such printed patterns for surface-enhanced Raman spectroscopic studies was also demonstrated. However, achieving efficient nanofabrication with the capability for continuous and arbitrary patterning of plasmonic nanostructures using a bubble-free printing approach remains highly desirable and a significant challenge. Recently, we have demonstrated a white-light based plasmonic nanostructure printing with superior performance.^35,36^ In our previous work, we presented a bubble-free optical printing method that utilizes optically generated thermal and scattering forces for the static, point-by-point assembly of plasmonic nanoparticles. This approach was successfully applied to create highly sensitive SERS-active spots on both planar substrates^22^ and specialized optical fiber tips.^23^ However, the method was limited to creating discrete, localized assemblies; translating the spot to achieve uniform, continuous patterns for broader applications, such as in microelectronics, remained a significant challenge. In this work, we illustrate the fabrication of arbitrary plasmonic patterns on transparent substrates and investigate the role of experimental conditions such as laser power and writing speed in the patterns formed. The key novelty of the present work is the successful transition from that static, spot-printing technique to a dynamic, continuous-writing process. In addition, variation in the electrical conductivity of the patterns printed under different experimental conditions was investigated, and the feasibility of such conducting patterns for electrothermal trapping of the biological cells was demonstrated. Moreover, the potential of the printing approach for surface-enhanced Raman spectroscopic studies of analytical molecules was investigated by printing the crystal violet dye molecule along with the nanoparticles. Interestingly, the printed structure exhibited good SERS activity with a consistent Raman signal across the printed structure, highlighting the uniformity of the printed line. To the best of our knowledge, this is the first report of fabricating arbitrary conducting lines on a transparent glass substrate without the use of laser-induced microbubbles and without the need for postprocessing such as annealing and illustrating their electrothermal and Raman spectroscopy applications.

## 2. Experimental

### 2.1 Chemicals and materials

AgNO_3_ (≥99.0%, Merck Life Science Private Limited), tri-sodium citrate dihydrate (≥99%, Merck Life Science Private Limited), and crystal violet (88%, Loba Chemie Private Limited) were used.

### 2.2 Synthesis and characterization of Ag nanoparticles

The Ag nanoparticle colloidal solution was prepared *via* the Lee and Meisel method.^37^ Briefly, 16.89 mg of AgNO_3_ was suspended in 50 mL of deionized water and boiled to 90 °C. Then 40 mM of trisodium citrate solution is added dropwise to the boiling solution and magnetically stirred until the color changed to golden yellow.

The optical absorption spectra of the solution were recorded using a UV/visible spectrophotometer (JASCO V650) and included a band centered at 423 nm.^22^

### 2.3 Experimental setup

The schematic illustration of the experimental setup used for the present study is shown in Fig. S1. A green laser operating at a wavelength of 532 nm is coupled with the microscope objective of an inverted microscope (Nikon eclipse Ti2, Japan) using beam expander-dichroic filter assembly and is then focused on the upper surface of the glass substrate kept over the sample stage of the microscope. The sample stage of the microscope is integrated with a motorized two-dimensional translational stage (Micro position controller, HOLMARC). The visualization of the focal plane is carried out using Kohler illumination, and a CCD camera (Nikon, DS-Fi3) equipped with a microscope is used to image the samples. The average laser power at the output of the microscope objective was measured using an integrating sphere (OPHIR, 3A-IS-V1 ROHS). A volume of 100 µL Ag nanoparticle colloidal solution is placed over the substrate followed by illumination using a laser. The programmed movement of the microscope stage at specific velocities results in the formation of continuous arbitrary patterns. For *I*–*V* measurements, Au gap electrodes were fabricated using shadow masking and thermal evaporation. For electrothermal trapping, a 20-micron gap electrode was achieved through shadow masking, photolithography (HO-LWS-PUV-MT), and thermal evaporation. However, the yeast cell viability after trapping is not investigated in the present study. Electrical characterization was performed using a Keysight B2901B source measurement unit. The Raman spectroscopy setup is combined with the printing system by coupling a Horiba spectrometer through optical components (Horiba Scientific, IHR320-Symphony II CCD assembly) as shown in Fig. S2.

## 3. Results and discussion

**Fig. 1:**
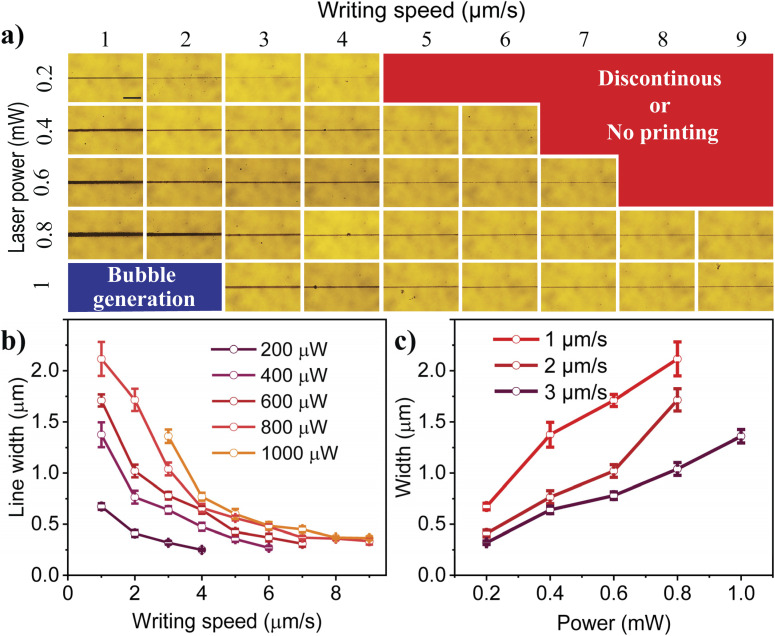
(a) Microscope images of the printed lines for various laser powers and writing speeds (scale bar 10 µm), (b) plot of the line width as a function of writing speed for various laser powers, and (c) plot showing the variation of line width as a function of laser power for various writing speeds.

In our study, the optical printing of plasmonic particles was performed by focusing a 532 nm laser onto the Ag colloidal solution placed on a microscope cover slide, which was mounted on a motorized sample stage. As discussed in our previous studies, the optical absorption of the plasmonic nanoparticles leads to Marangoni flow and the movement of particles to the irradiation zone and immobilization *via* van der Waals interaction.^22^ To achieve continuous printed patterns, the stage was programmed to move in a predetermined direction using the stage control software while the Ag colloidal droplet was exposed to the laser. A systematic approach was undertaken to achieve continuous patterns using laser powers of 0.2–1 mW and stage scanning speeds (writing speed) of 1–9 µm s^−1^. The printed patterns imaged through an optical microscope are shown in Fig. 1a. Interestingly, we were able to achieve a continuous printed line at a laser power of 0.2 mW and a writing speed of 1 µm s^−1^. The line width was measured to be 0.7 µm, highlighting the submicron patterning capability of the printing technique at a very low excitation power. An increase in the writing speed led to a reduction in both line width and thickness (observed from the variation in opacity), with a minimum width of 0.25 µm achieved at a printing speed of 4 µm s^−1^. Beyond this speed, no observable pattern was detected, which may be attributed to the rapid shifting of the thermal gradient along the writing direction. At slower writing speeds, sufficient time is available for thermal convection to deposit nanoparticles in a continuous manner.^37,38^ In contrast, higher speeds cause the local thermal convection region to shift rapidly, reducing the available time for deposition, which in turn decreases the line width and thickness. At excessively high writing speeds, the shift becomes so rapid that the printing process fails altogether. Furthermore, at a given writing speed, an increase in laser power was observed to result in greater line width and thickness. Notably, higher laser power also extended the range of achievable writing speeds. As shown in the figure, the writing speed range for 0.2 mW was limited to 1–4 µm s^−1^, while increasing the power to 0.4 mW expanded the range to 1–6 µm s^−1^. At 0.6 mW, the range further increased to 1–7 µm s^−1^. Interestingly, at 0.8 mW, successful printing was achieved across the entire speed range, from 1 to 9 µm s^−1^. However, further increasing the laser power resulted in bubble formation, causing discontinuities in the printing structure. As shown in Fig. 1a (and Fig. S3), at 1 mW laser power, writing speeds below 2 µm s^−1^ resulted in bubble formation. This phenomenon can be attributed to the increased laser power generating higher temperatures, which, in turn, resulted in solvent evaporation and bubble formation. Nevertheless, this issue can be mitigated by increasing the writing speed, thereby reducing the effective temperature and enabling successful printing.

A close probing of the line width of the continuously printed patterns from the optical microscopic images clearly illustrated that, for a given laser excitation power, the line width of the printed pattern decreases with an increase in writing speed (Fig. 1b). The variation in line width was significant at lower writing speeds (1 µm s^−1^ to 2 µm s^−1^) but saturated as the laser writing velocity increased. This effect is more evident for the patterns printed at higher laser powers. Moreover, at lower writing speeds, the laser power was found to have a pronounced effect on the line with of the printed pattern. At higher laser powers (0.8 and 1 mW) and writing speeds (>7 µm s^−1^), the linewidth was found to be nearly the same. On the other hand, for a given writing speed, the line width of the continuously printed pattern was observed to increase with laser power (Fig. 1c). In addition, large-area printing and print consistency are demonstrated in Fig. S4, with a line width variation of less than 6% along a 1 mm printed line.

**Fig. 2:**
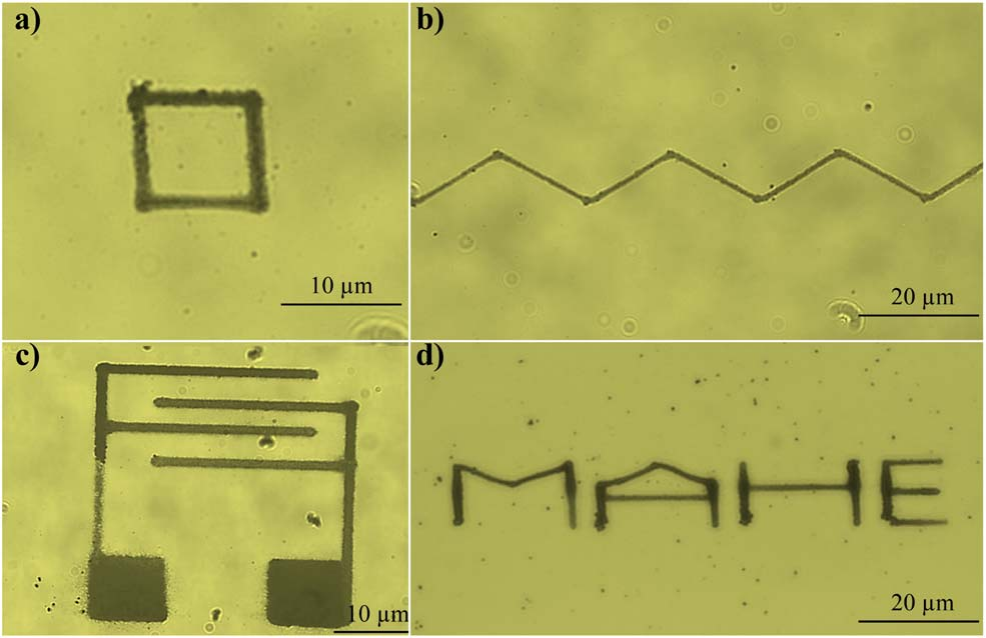
Laser-induced arbitrary pattern of Ag nanoparticles; (a) a square, (b) a triangular wave, (c) an interdigitated electrode, and (d) the word “MAHE”.

For effective nanofabrication, it is essential to achieve arbitrary patterning capabilities. In this study, the ability of the proposed printing technique to deposit plasmonic particles in desired shapes and designs was further explored by programming the movement of the sample stage. Initially, a 10 µm square pattern and a triangular wave pattern were attempted and successfully fabricated, as shown in Fig. 2a and b. Furthermore, for microelectronics applications, the realization of electrode patterns is crucial. Here, we demonstrate the fabrication of a sophisticated interdigitated electrode pattern (Fig. 2c), highlighting the potential of this technique for microelectronics. To further showcase intricate arbitrary patterning, the word “MAHE” was successfully written, as shown in Fig. 2d. This study unambiguously demonstrates the potential of the bubble-free laser-assisted printing approach to create arbitrary-shaped patterns for advanced applications in nanofabrication and microelectronics.

**Fig. 3:**
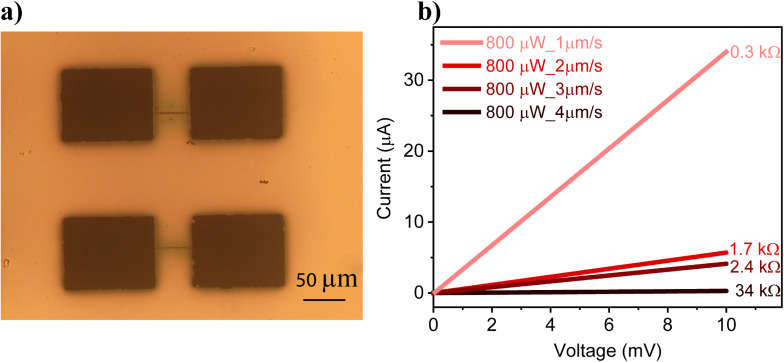
(a) The printed Ag lines across a 50 µm gap electrode. (b) The *I*–*V* characteristics of Ag conducting lines printed at 800 µW power and a writing speed of 1–4 µm s^−1^.

After achieving continuous patterns without bubble formation, the connectivity of the printed pattern was validated by measuring its electrical conductivity. To accomplish this, laser patterning was performed on prefabricated gold electrodes obtained through shadow masking and thermal evaporation. Ag lines were printed across 50 µm gap electrodes at different speeds using a laser power of 800 µW. An optical image of a printed device is shown in Fig. 3a. The *I*–*V* characteristics of the devices were measured in the voltage range of 0 to 10 mV, and the current was recorded using a source measure unit. Interestingly, the printed patterns exhibited conductivity, with conductance increasing as the writing speed decreased, as shown in Fig. 3b. The resistance of the line decreased from 34 kΩ at a writing speed of 4 µm s^−1^ to approximately 0.3 kΩ at a writing speed of 1 µm s^−1^. Typically, achieving conductive lines through optical printing methods requires a post-sintering process (thermal or chemical). In contrast, in this study, conducting patterns were achieved without any post-processing. This is highly advantageous for nanofabrication and microelectronic applications, as it enables the direct printing of conducting metal patterns, eliminating the need for complex and sophisticated lithography processes. The observed conductivity may be attributed to the coalescence of nanoparticles during the printing process, which creates electrically conducting pathways for electrons. Slower writing speeds allow more time for nanoparticle coalescence, thereby enhancing conductivity, as evidenced by the *I*–*V* characteristics. Additionally, laser power plays a significant role in the packing of nanoparticles during printing. For a given writing speed, increasing the laser power promotes greater coalescence, which in turn increases the electrical conductance. This is supported by printing conducting lines at 600 µW and 800 µW, both at a writing speed of 1 µm s^−1^. As shown in Fig. S5, the electrical conductance is significantly higher at higher laser power.

**Fig. 4:**
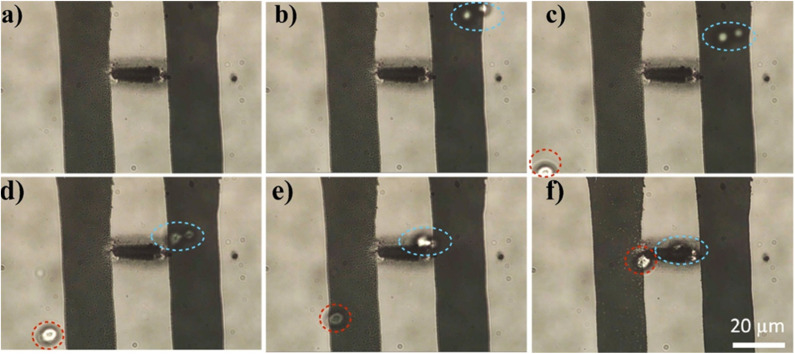
(a) The printed conducting line of resistance ∼1 kΩ between the gap electrode and (b–f) sequences from the electrothermal trapping event of the yeast cells (at a voltage of 1 V).

The ability to directly print conducting patterns without any post-processing enabled us to fabricate an electrothermal trapping platform. Trapping is a technique used to immobilize and study individual or few particles under various conditions, with widespread applications in materials science, nanotechnology, and medical sciences.^39^ Unlike conventional optical trapping, which relies on bulky complicated experimental setups, electrothermal trapping offers the advantages of miniaturization, CMOS compatibility, and potential integration into lab-on-a-chip platforms. To demonstrate the capability of the present fabrication technique for developing an electrothermal trapping platform, a conducting line with a resistance of approximately 1 kΩ was printed between the gap electrodes, as shown in Fig. 4a. The resistance of the printed line was deliberately designed to be higher than that of the electrodes to obtain a more pronounced joule heating effect in the printed region, facilitating particle trapping. A 100 µL solution of yeast cells was placed onto the platform, and a voltage of 1 V was applied across the electrodes. This resulted in an electrical current of approximately 1 mA through the line, generating joule heating and inducing thermal convection flow, which caused the yeast cells to migrate toward the line. Within 100 seconds, the cells were successfully trapped in the printed region, as shown in Fig. 4b–f. With time, more yeast cells get trapped, as shown in Fig. S6. Importantly, the printed pattern remained stable in the presence of the water solution, demonstrating the robustness of the fabricated platform. To further demonstrate the stability of the printed patterns, the structures were subjected to various mechanical stress conditions, including ultrasonication (Fig. S7), he Scotch tape peel test (Fig. S8), and the scratch test (Fig. S9). The results show that the printed patterns remained stable under ultrasonication and Scotch tape peel forces. However, the structures could not withstand the scratch test, which is consistent with the behaviour observed in thermally evaporated, lithographically patterned Ag structures.

**Fig. 5:**
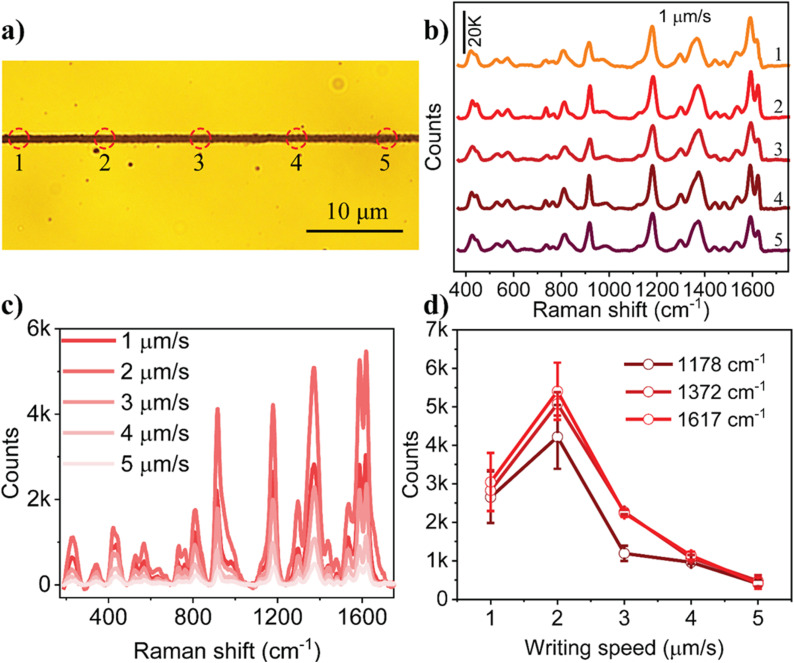
(a) Five different locations along the Ag line, printed at a writing speed of 1 µm s^−1^, from which the Raman spectra of crystal violet (100 µM) dye were measured with an excitation power of 500 µW, and (b) the corresponding spectra from these locations. (c) Raman signals of CV (1 µM) obtained from lines printed at different writing speeds, ranging from 1 µm s^−1^ to 5 µm s^−1^ (at an excitation power of 500 µW and exposure time of 10 s). (d) Variation in the intensities of Raman bands (1178 cm^−1^, 1372 cm^−1^, and 1617 cm^−1^) as a function of writing speed.

The potential of optical printing as a platform to carry out surface-enhanced Raman scattering studies was evaluated by printing the particles along with crystal violet (CV) molecules. Herein, a colloidal solution consisting of Ag and CV in a 9 : 1 ratio was excited. The optical absorption of the particles generated thermal convective flow, leading to the printing of Ag nanoparticles along with the dye molecules. The plasmonic field generated by the printed Ag nanoparticles under 532 nm excitation enabled the collection of surface-enhanced Raman signals from the printed CV molecules.^40–42^ The Raman signals of CV were measured at five different locations along the printed line, which was fabricated at a writing speed of 1 µm s^−1^ (Fig. 5a), showing nearly identical intensities (Fig. 5b). Additionally, Ag printing was performed with a fluorescent dye, and fluorescence microscopy further confirmed the uniformity of the printed line (Fig. S10). This consistency confirms the continuity and uniformity of the printed line. To demonstrate the SERS activity, an Ag line was printed at a power of 400 µW with varying writing speeds ranging from 1 to 5 µm s^−1^. Subsequently, 10 µL of CV (1 µM) was deposited and dried onto the printed Ag lines. Raman spectra of CV were then collected from five different spots along the conductive lines to evaluate the enhancement effect. As depicted in Fig. 5c, the Raman signal from the line printed at 2 µm s^−1^ exhibited a significantly higher enhancement compared to the other lines. We hypothesize that at a speed of 1 µm s^−1^, the gaps between the printed particles were too narrow for the molecules to occupy effectively. Meanwhile, at speeds ranging from 3 to 5 µm s^−1^, the gaps became too wide, preventing the formation of an optimal gap for intense hotspots, which resulted in a reduced enhancement. The study illustrated that a plasmonic line printed at 2 µm s^−1^ can be considered as the optimized pattern for Raman spectroscopic studies of analytes. The variations in the intensity of prominent peaks of the CV centred at 1178, 1372, and 1617 cm^−1^ are given in Fig. 5d. All these spectral peaks exhibit maximum intensity for the plasmonic printed line fabricated at a writing speed of 2 µm s^−1^. Additionally, the co-printed lines were subjected to ultrasonication for 20 minutes, and their SERS signals were measured. As shown in Fig. S11, there is no significant variation in signal intensity before and after sonication, indicating good structural robustness of the print under mechanical agitation. A comparison table (Table S1) in the SI summarizes the performance parameters of our method with state-of-the-art fabrication techniques reported in the literature, while a quantitative comparison between our bubble-free method and the previously reported bubble printing technique for pattering Ag nanoparticle lines is provided in Table S2.

## 4. Conclusion

In summary, we developed a bubble-free optical printing approach to achieve continuous patterning of plasmonic nanoparticles and optimized key experimental parameters, such as laser writing speed and power, to create patterns suitable for both electrical and optical applications. By tailoring the writing speed and laser power, we were able to achieve continuous Ag plasmonic patterns, with a line width that decreased with the writing speed and increased with laser power. The *I*–*V* characteristics of the printed lines clearly demonstrated that by tuning the parameters (writing speed and laser power) the electrical properties of the lines could be effectively controlled and optimized. Furthermore, the potential of the printed line for electrothermally trapping biological cells such as yeast cells was demonstrated by applying a voltage of 1 V across a conducting channel that exhibits a resistance of 1 kΩ. In line with earlier studies of optically printed plasmonic substrates, our printed lines also exhibited excellent Raman signal enhancement and similar signal count along the printed region thus illustrating the uniform plasmonic particle printing capability of the approach. It was also observed that the Raman signal enhancement depends upon the laser writing speed (power) and in our case 2 µm s^−1^ (400 µW) was found to be the optimum for the Raman signal enhancement. Our direct optical printing offers a straightforward, cost-effective, and facile method for printing plasmonic particles of arbitrary patterns and enables the realization of advanced electronic and optical sensing platforms.

## Conflicts of interest

The authors declare no conflict of interest.

## Supplementary Material

NA-007-D5NA00742A-s001

## Data Availability

Data will be available from the corresponding author upon request. Supplementary information is available. See DOI: https://doi.org/10.1039/d5na00742a.
