# Supplementary material for: Continuous bubble-free laser printing of plasmonic nanostructures enabling annealing-free ohmic conduction and multifunctional trapping/spectroscopy studies
Source: Nanoscale Adv. 2025 Oct 29;7(24):8085–92. doi: 10.1039/d5na00742a (PMC12608513; doi:10.1039/d5na00742a)
Supplement: NA-007-D5NA00742A-s001 [file NA-007-D5NA00742A-s001.pdf]

Supplementary Information (SI) for Nanoscale Advances.
This journal is © The Royal Society of Chemistry 2025

Supporting Information.

Continuous bubble-free laser printing of plasmonic nanostructures enabling annealing-free ohmic conduction and multifunctional trapping/spectroscopy studies

Monisha K,[a] Bharath Bannur,[b] Shreyas MS,[b] Sajan D. George*[b, c]

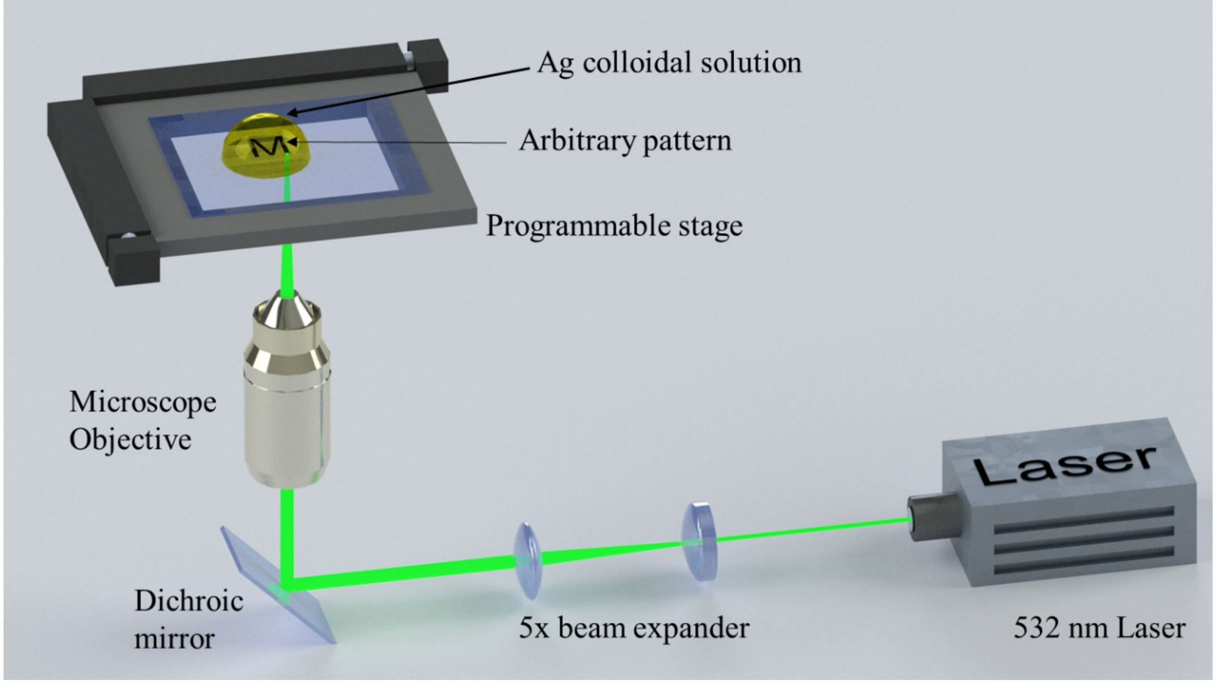

Figure S1: The schematic illustration of the experimental setup.

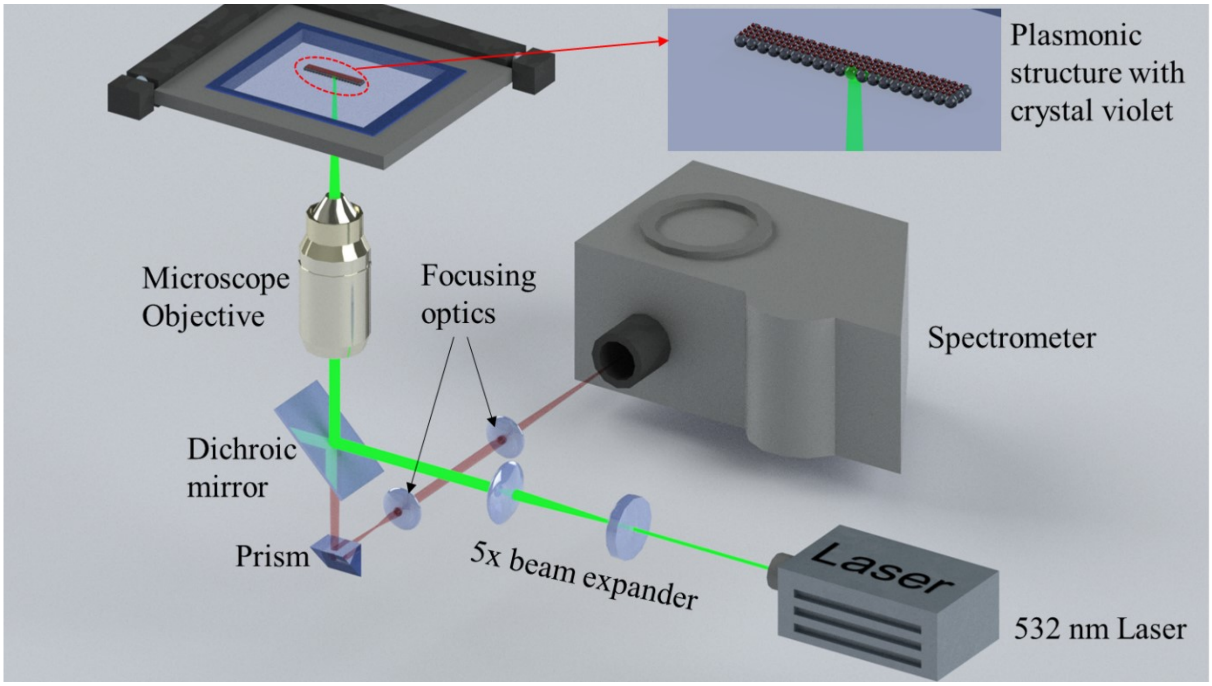

Figure S2: The schematic illustration of the printing setup coupled with spectrometer for SERS studies.

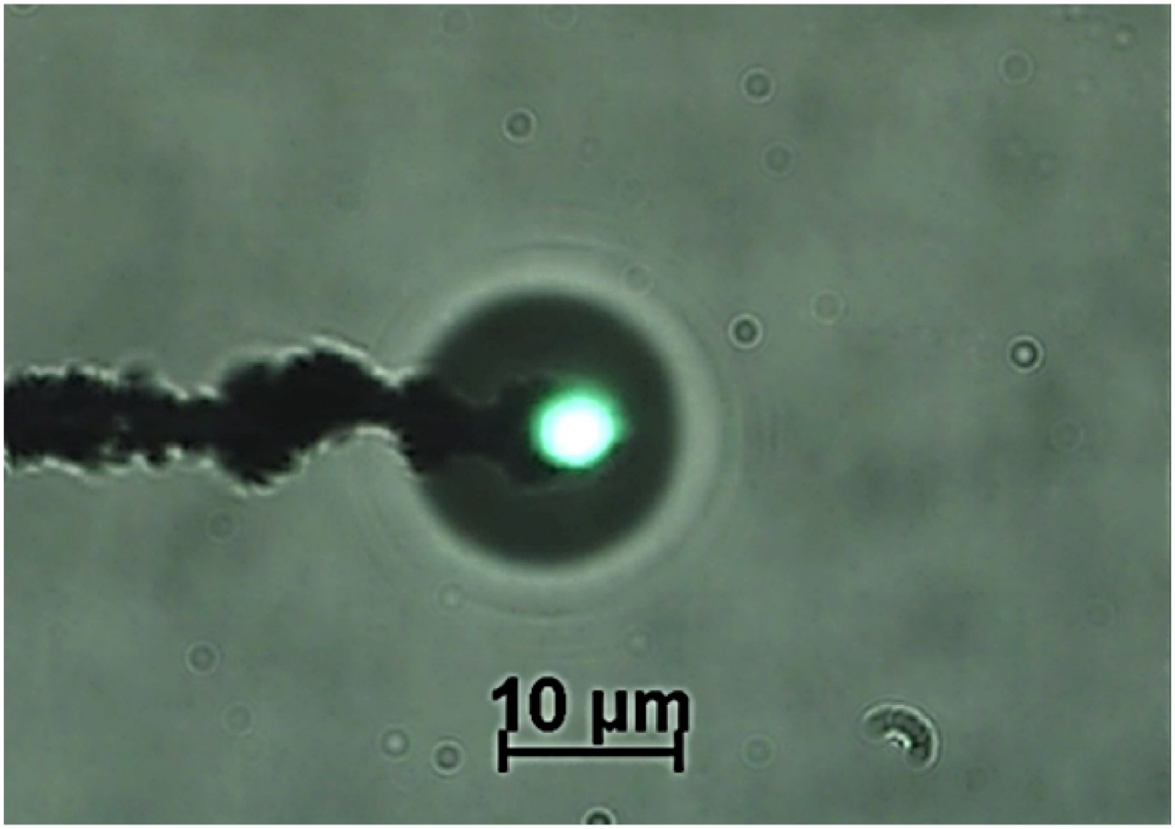

Figure S3: Formation of microbubble at a laser power of 1 mW with writing speed 1 $\mu m/s$.

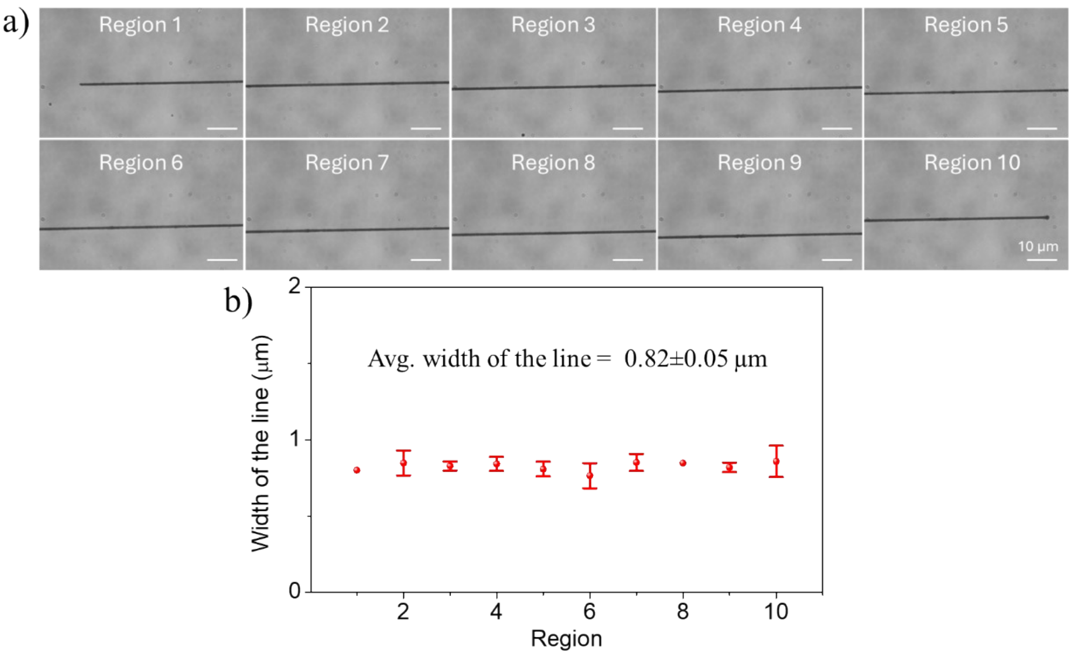

Figure S4: (a) Optical images from different regions along the 1 mm printed line, demonstrating uniform deposition; (b) line width measurements at various regions, showing a variation as low as 6%. These results collectively confirm the consistency of the printing process over a large scale.

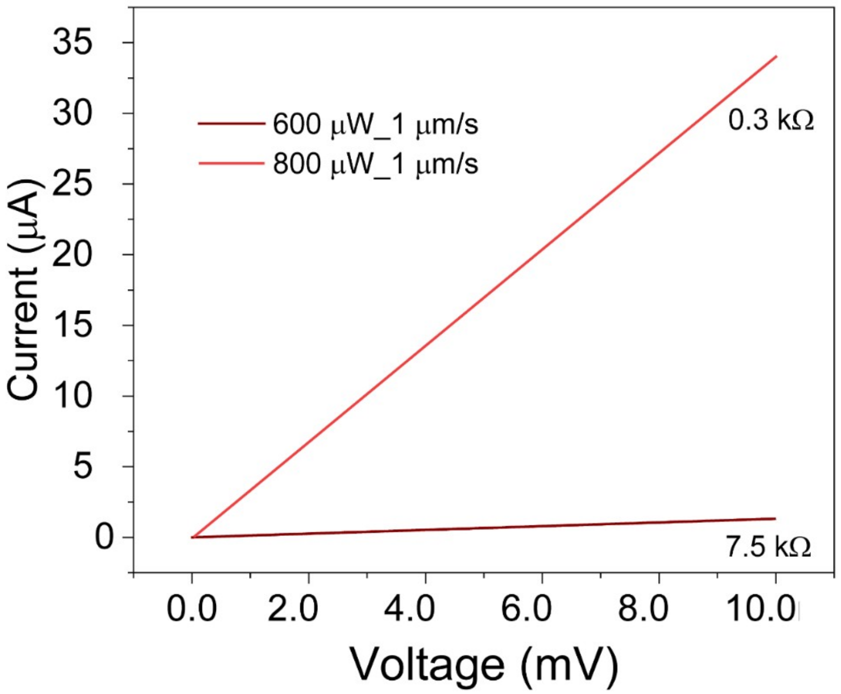

Figure S5: The I-V characteristics of Ag conducting lines printed at 800 μW and 600 μW power and writing speed of 1 μm/s.

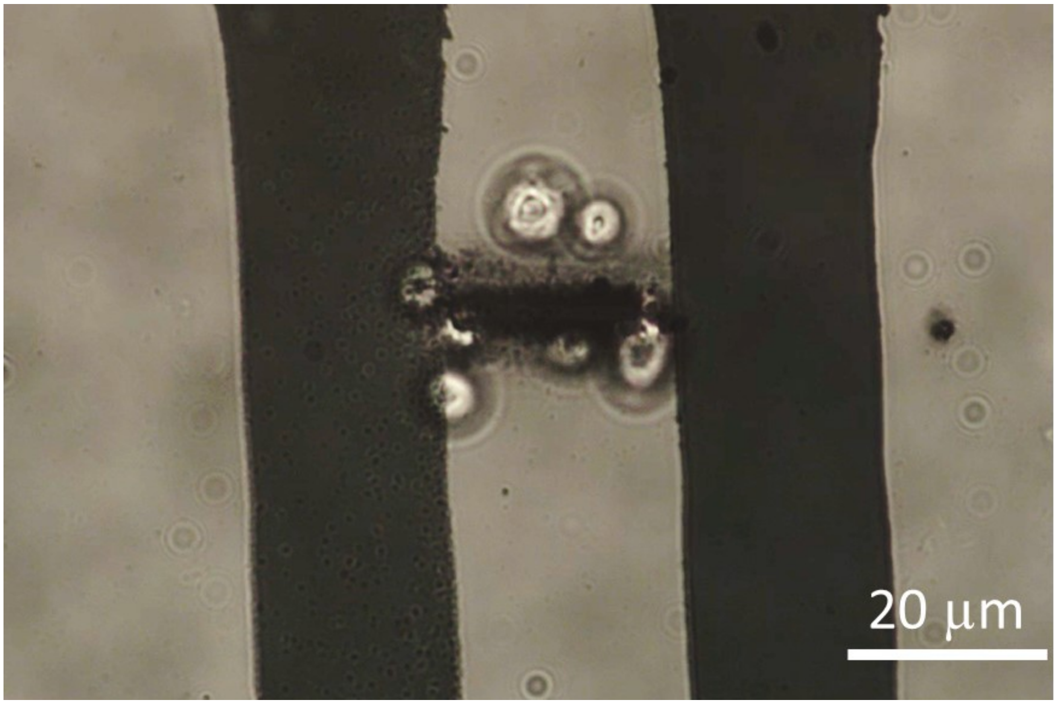

Figure S6: Assembly of yeast cells.

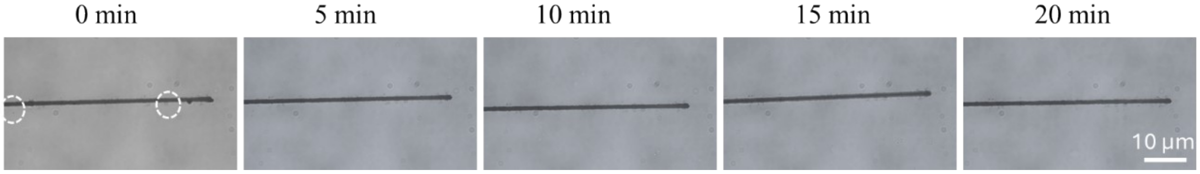

Figure S7: The printed line was subjected to ultrasonication for varying durations. The pattern remained stable for 20 minutes of sonication, demonstrating the robustness of the printed structure. However, stray deposited particles were removed during sonication, as indicated by the dotted regions.

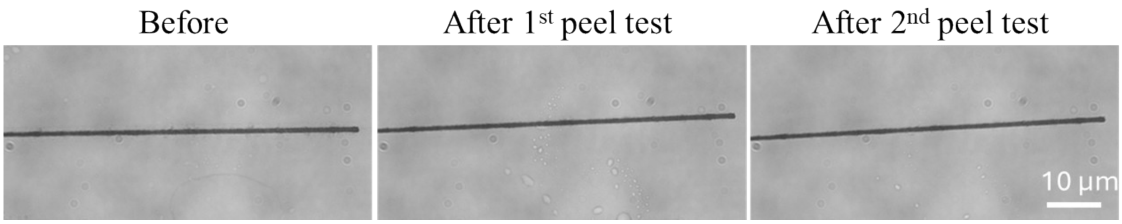

Figure S8: Scotch tape peel test performed to evaluate the mechanical robustness of the printed pattern. As shown, the printed line remained adhered to the substrate even after two peel cycles, indicating good adhesion.

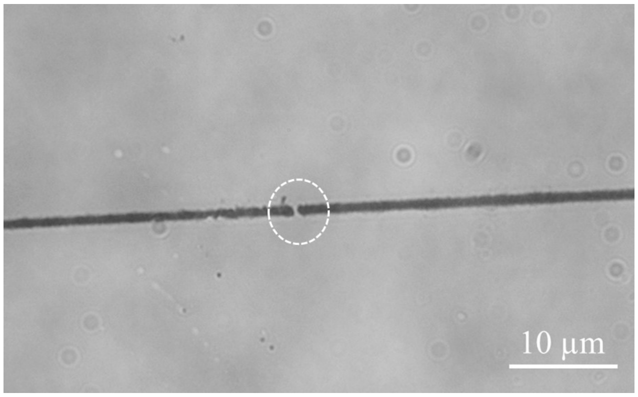

Figure S9: Scratch test performed on the printed line using a probe needle. As observed, the printed structure could not withstand the applied force and was peeled off, as indicated by the dotted region.

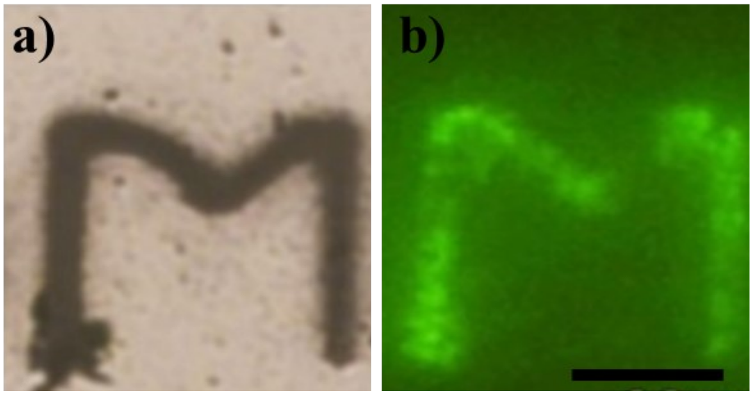

Figure S10: a) Optical microscope image of the Ag print along with acridine orange dye, b) Fluorescence image of the pattern showing the uniformity of the print without any double-hump profile. (scale bar 10 μm)

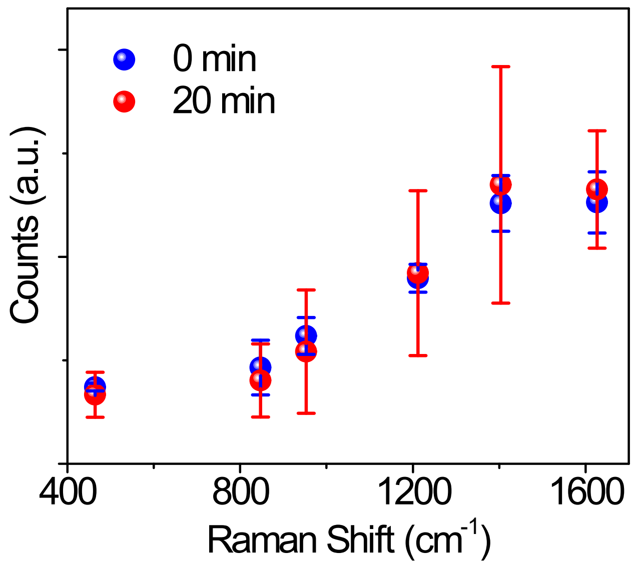

Figure S11: To evaluate the structural integrity and functional stability of the printed pattern, crystal violet and Ag nanoparticles were co-printed, and SERS measurements were conducted before and after sonication. Intensity variation of major peaks shows no significant change after 20 minutes of sonication, demonstrating the structural and functional integrity.

Table S1: Comparison of performance parameters (resolution and writing speed) of various techniques with our work.

Work	Resolution	Maximum writing speed	Remarks	Reference
**Bubble free optical printing plasmonic structure**	~250 nm	9 µm/s	Simple setup<br>Low-laser power printing<br>Direct metal printing	This work
**Fabrication of polyaniline microstructure using laser induced microbubble**	~ 650 nm	0.8 mm/s	Bubble-induced printing,<br>Complex set up,<br>Ability to print various materials,<br>High temperature generation may affect the soft materials,	[1]
**Bubble printing of nanoparticle assembly**	-	1.2 mm/s		[2]
**Bubble printing of $Ti_3C_2T_X$ MXene**	1000 nm	652 µm/s		[3]
**Fabrication of Ag conducting line**	1700 nm	9 mm/s		[4]

**Bubble printing of quantum dots**	~ 650 nm	10^{-2} m/s		[5]
**Fabrication of CdS nanostructure using electron beam lithography**	~ 4 nm	-	Complex and sophisticated system	[6]
**Patterning of diamond by electron-beam-induced etching**	10 nm	-	Require sophisticated systems	[7]
**Direct laser writing through multiphoton and peripheral photoinhibition photolithography**	100 nm	1000 µm/s	Require sophisticated setup (consists of Femtosecond and Picosecond Lasers, Beam Modulation and Synchronization, Photoresists etc.)	[8]
**3D electron-beam writing using spider silk as a resist**	Sub-15 nm	-	Require sophisticated setup,<br>Multistep approach.	[9]
**Direct photo-Patterning of efficient and stable quantum dot light-emitting diodes**	Microscale	-	Multistep process, Complex	[10]
**Direct X-ray and electron-beam lithography of halogenated zeolitic imidazolate frameworks**	Sub-50 nm	-	Complex	[11]

Table S2: Comparison of the present bubble-free method with bubble printing technique for patterning conducting Ag nanoparticle lines.

Performance Metric	**Bubble-Free laser printing of Ag nanoparticles (This work)**	**Laser-Driven Bubble Printing of Ag nanoparticles** [4]
**Minimum Line Width (nm)**	250	1700
**Laser Intensity (mW)**	0.2-0.8	32
**Writing Speed (µm/s)**	10	30

**Electrical Resistance, ohms (without annealing)**	~ 300	~500 (calculated)
**Laser frequency modulation**	Not required	Required
**Applications demonstrated**	Conducting line, Electrothermal Trapping, SERS	Conducting line
**Advantages**	Simple, low laser power, bubble-free low temperature, high resolution	High speed

References

[1] E. Edri, N. Armon, E. Greenberg, E. Hadad, M. R. Bockstaller, H. Shpaisman, *ACS Appl. Mater. Interfaces,* 2020**, 12**, 22278-22286.

[2] E. H. Hill, C. Goldmann, C. Hamon, M. Herber, *Phys. Chem. C*, 2022, **126**, 7622-9.

[3] M. Herber, D. Lengle, S. R. Valandro, M. Wehrmeister, E. H. Hill, *Nano Lett.* 2023, **23**, 6308-6314.

(4) N. Armon, E. Greenberg, M. Layani, Y. S. Rosen, S. Magdassi, H. Shpaisman, *ACS Appl. Mater. Interfaces*, 2017, **9**, 44214-44221.

[5] B. Bangalore Rajeeva, L. Lin, E. P. Perillo, X. Peng, W. W. Yu, A. K. Dunn, Y. Zheng, *ACS Appl. Mater. Interfaces,* 2017, **9**, 16725-33.

[6] M. S. Saifullah, M. Asbahi M, D. C. Neo, Z. Mahfoud, H. R. Tan, S. T. Ha, N. Dwivedi, T. Dutta, S. bin Dolmanan, Z. Aabdin, M. Bosman, *Nano Lett.* 2022, **22**, 7432-40.

[7] V. Dergianlis, M. Geller, D. Oing, N. Wöhrl, A. Lorke. *Nanotechnology*. 2019, **30**, 365302.

[8] X. Liu, Q. Liu, M. Luo, L. Xu, C. Kuang, X. Liu, *Opt. Lett*. 2025, **50**, 1675-8.

[9] N. Qin, Z. G. Qian, C. Zhou, X. X. Xia, T. H. Tao, *Nat. Commun*. 2021, **12**, 5133.

[10] Z. Fu Z, L. Zhou, Y. Yin, K. Weng, F. Li, S. Lu, D. Liu, W. Liu, L. Wu, Y. Yang, H. Li. *Nano Lett*. 2023, **23**, 2000-8.

[11]M. Tu, B. Xia, D. E. Kravchenko, M. L. Tietze, A. J. Cruz, I. Stassen, T. Hauffman, J. Teyssandier, S. De Feyter, Z. Wang, R. A. Fischer, A. *Nat. Mater.* 2021, 20, 93-9.
